# A scalable dissemination model for training faculty in mentor development and networking

**DOI:** 10.18103/mra.v14i5.7423

**Published:** 2026-05-27

**Authors:** Akshay Sood, Beth Tigges, Deborah Helitzer

**Affiliations:** 1 University of New Mexico Health Sciences Center, Albuquerque, NM, USA; 2 Arizona State University College of Health Solutions, Phoenix, AZ

**Keywords:** Mentor development, mentoring network, theory of planned behavior, ECHO, knowledge, perceived behavioral control, developmental networks, faculty training

## Abstract

Faculty success in academia depends on effective mentoring and developmental networks, yet shortages in trained mentors, limited networking skills and inconsistent mentoring quality impact career advancement. While structured mentor development programs improve mentor competency and curriculum-based training improves networking self-efficacy, these programs are widely unavailable and/or underutilized. To address this gap, we evaluated scalable, evidence-based faculty training programs disseminated to academic institutions across the United States using the Project ECHO (Extension of Community Health Outcomes) telementoring model. Two interventions—Mentor Development and Mentoring Networks—were implemented from 2023 to 2025. Participation, demographics and reach were tracked, while surveys assessed knowledge, content relevance, intent to apply knowledge, communication, barriers and perceived impact. Knowledge gains were measured by comparing retrospective pre/post- test scores on a Likert scale. A total of 821 Mentor Development and 556 Mentoring Networks attendances were recorded, with mean attendance of 20.5 and 15.4 unique individuals, respectively. Both programs showed significant increases in knowledge (p<0.001; Cohen's d = 1.3 and 1.7, respectively). Participants reported strong intentions to apply the content (means = 4.6, 4.6), high relevance (means = 4.0, 3.9), and improved communication and collaboration within teams (means = 4.2, 4.4). Reported impacts included improved knowledge (84–94%), skills (59–61%), and performance (32–36%), with limited time and resources as the most common barriers. The Project ECHO model is a feasible, scalable approach for disseminating mentor development and networking training to strengthen academic workforce capacity and reduce inequities across diverse, real-world academic settings.

## Introduction

Effective mentoring is a critical determinant of faculty success in academic medicine, influencing career advancement, research productivity, and professional satisfaction. However, persistent challenges—including shortages of trained mentors, variability in mentoring quality, and limited faculty networking skills—continue to constrain faculty development.^1^ Mentoring competencies are often acquired informally, through role modeling and experience rather than through structured, competency-based training,^2^' ^3^ which contributes to inconsistencies in mentoring effectiveness.

Structured mentor development programs have demonstrated efficacy in improving mentoring competencies. Randomized controlled trials and quasi-experimental studies show that competency-based training can enhance mentors' knowledge, skills, and confidence, with effects sustained over time.^4–7^ In addition, the availability of formal mentor training programs is a key component of a positive organizational mentoring climate, particularly valued by women and early-career faculty.^8^' ^9^ Despite this evidence, adoption of such programs remains limited; the number of institutions offering formal training is limited, and even when they are available, awareness and participation in these programs are suboptimal.^8–10^ These gaps highlight the need for scalable and accessible approaches to mentor development.

The Project ECHO (Extension for Community Healthcare Outcomes) model offers a potential solution by enabling the dissemination of expertise through a virtual, hub-and-spoke telementoring framework. Originally developed to improve chronic disease management,^11–13^ the model has been successfully adapted for education and workforce development, including mentor and network training.^14–17^

The ECHO model is grounded in five core principles. First, it leverages technology such as multipoint video-conferencing and the Internet to maximize scarce resources. Second, it utilizes an internet-based data system (i.e., iECHO software [Hangzhou IECHO Science & Technology Co., Ltd.]) to track and monitor outcomes. Third, the model seeks to reduce variation in training processes while facilitating the dissemination of best practices. Fourth, it emphasizes case-based learning through structured dialogue among experts and peers. Finally, and most central to the present work, the ECHO model aims to build a digital community of learning that prioritizes reciprocity in the exchange of knowledge and skills, fosters trust and mutual respect, and recognizes that all participants contribute valuable expertise, thereby supporting contextualized and collaborative learning.^18^ This approach is consistent with established educational theories, including social cognitive theory and situated learning, and emphasizes active participation and experiential learning.^19–21^

Given the relational and context-dependent nature of mentoring, program outcomes may be understood within the framework of the Theory of Planned Behavior (TPB).^22^ This theory posits that behavior is driven by intention, which is shaped by attitudes, subjective norms, and perceived behavioral control. In academic settings, these constructs are likely influenced by institutional culture and mentoring climate, which may moderate the translation of intention into mentoring behavior.

This study aimed to evaluate the outcomes of scalable, ECHO-based mentor development and networking training programs implemented across multiple academic institutions across the United States (US). Specifically, we assessed faculty participation, changes in knowledge and mentoring competencies, and perceived program impact.

## Methods

### STUDY DESIGN AND SETTING

We conducted a retrospective observational program evaluation of two virtual synchronous faculty development programs delivered via the ECHO model: the Mentor Development program (August 2023—September 2025) and the Mentoring Networks program (October 2023-November 2025).

### ECHO-BASED PROGRAM DELIVERY

Curricula for both programs, shown in Tables 1 and 2, were competency-based and iteratively refined using structured instructional design methods.^23^

Both programs followed a standard format for ECHO sessions, emphasizing interaction more than didactic content. Sessions incorporated: (i) a brief introduction/announcement; (ii) short expert didactic/s with facilitated Q&A; and (iii) multiple interactive case-based discussions to contextualize concepts and enable peer learning. The programs were offered free of charge and continuing education credit was available at no cost, contingent on completion of the post-session survey.

### PARTICIPANTS

Eligible participants were faculty who attended one or more sessions of either program during the evaluation period. The initial higher education institutions where the programs were disseminated were in the Southwest and Mountain West regions of the US. These institutions had grant-based relationships with the University of New Mexico Health Sciences Center to jointly develop mentoring infrastructure. Participants represented diverse institutions, disciplines, and career stages, engaged voluntarily, and participated amid competing professional demands. No honoraria were provided for participation.

Attendance was summarized as total attendance (non-unique) and the average number of unique participants per session. Professional background was self-reported (physician, nurse/nurse practitioner, pharmacist, social worker, registered dietician, or PhD/other/unknown). Participation in evaluations and requests for continuing education credits was voluntary.

### DATA SOURCES AND DATA MANAGEMENT

Both programs used the iECHO software as the outcomes and continuing education credit management system to track attendance, administer evaluations, and process continuing education credits. Program evaluation surveys were administered electronically, and REDCap—a secure, web-based platform—was used for data capture and management.^24^

### MEASURES

#### Primary learning outcome (knowledge):

Self-reported knowledge was measured with a retrospective pre-test/post-test at the end of the sessions, using a 5-point Likert scale (1 : 'not at all knowledgeable' to 5: 'extremely knowledgeable').

#### Application/competence and relevance:

Post-session items assessed participants' intention to use session content, intention to apply skills in teams, perceived relevance to current work, and perceived improvement in communication/collaboration (5-point Likert scales aligned to each construct).

#### Self-reported impact and barriers:

Participants indicated whether the session increased/improved their knowledge, skills, or performance, or whether it produced no change. They also selected barriers to applying the session content.

#### Continuing education credits:

For each program, we summarized the credit type (CME, CEU, CE, CNE), the number of attendees requesting credit, and the total credit hours awarded.

### STATISTICAL ANALYSIS

We calculated descriptive statistics for attendance, evaluation response rates, and continuing education credits. For outcomes, we report means and standard deviations. Because individual-level pairing was not available for knowledge ratings, we compared retrospective pre- vs. post-scores within programs using unpaired t-tests, with two-sided α=0.05. Cohen's d was used to estimate standardized effect sizes with conventional benchmarks. All analyses were conducted at the program level. A p-value of <0.05 was considered statistically significant.

### ETHICAL CONSIDERATIONS

This project analyzed routinely collected educational program evaluation data for quality improvement; data were aggregated and de-identified before analysis. Institutional Review Board (IRB) approval was obtained (HRPO ECHO for Education IRB protocol 18–024).

## Results

From August 2023 to September 2025, the Mentor Development program delivered 40 sessions across five courses, with a total of 821 attendances and a mean attendance of 20.5 unique individuals per session (Table 3). Evaluation response rates averaged 37.8% of all faculty participants. From October 2023 to November 2025, the Mentoring Networks program delivered 36 sessions over five courses with 556 total attendances, averaging 15.4 unique individuals per session and a 21.0% evaluation response rate.

Among faculty participants, physicians represented 23% and 21% of the Mentor Development and Mentoring Networks programs, respectively (Table S1). Nurses and nurse practitioners accounted for 10% and 8%, respectively, in the two programs. The largest category of participants was mixed PhD and Other/Unknown (61% Mentor Development; 66% Mentoring Networks). The Mentor Development program awarded 236 total educational credit hours, whereas Mentoring Networks awarded 91.5 hours (Table S2). CME was the most frequently requested credit type for both programs.

Higher education institutions from New Mexico, Arizona, and Oklahoma in the Southwestern US accounted for the highest level of faculty participants, including representation from sites within the Navajo Nation (Figures 1 and 2).

While participants came from multiple organizations across New Mexico, including the Navajo Nation, the majority were affiliated with the University of New Mexico Health Sciences Center. Additional participants joined from Hawaii, Nevada, Colorado, Idaho, Ohio, and North Carolina, USA.

Both programs demonstrated statistically significant improvements in self-reported knowledge (p<0.001 for both analyses, Table 4). Knowledge for Mentor Development participants increased from a mean score of 2.6 (retrospective pre-test) to 3.6 (post-test); Mentoring Networks participants' scores increased from 2.3 to 3.5, respectively. Standardized effect sizes were Cohen's d = 1.3 and 1.7 for the Mentor Development and Mentoring Networks programs, respectively.

Most participants reported strong intentions to apply session content in their work (mean = 4.6 for both programs; Table 5). Participants also reported strong intentions to apply knowledge/skills in a team environment (Mentor Development mean=4.4; Mentoring Networks mean=4.5) and improved ability to communicate/collaborate in teams, with mean ratings of 4.2 (Mentor Development) and 4.4 (Mentoring Networks). Relevance to work ratings were high (Mentor Development mean=4.0; Mentoring Networks mean=3.9).

The impact of training on various educational outcomes is shown in Table 6. Mentor Development participants and Mentoring Networks participants reported knowledge gains of 84% and 94%, respectively. A positive impact on skills and performance was reported by 59% and 61%, and 32% and 36%, respectively (Table 6).

Whereas 64% of respondents from the Mentor Development program and 43% of respondents from the Mentoring Networks program reported no barriers to using the content, the need for additional training and the lack of time and resources were the most frequently reported by those individuals who did report barriers to using the content (Table 7). No participants described commercial bias in the sessions.

## Discussion

Prior randomized controlled trials (RCTs) have established the efficacy of a structured mentor development program under controlled conditions, demonstrating large and sustained improvements in mentoring competency.^7^. Those studies answered the question of whether these interventions can work when delivered with high fidelity, protected time, and carefully selected participants. In contrast, the present study provides evidence of effectiveness, demonstrating that these same evidence-based programs do work when scaled and implemented in real-world academic settings using the Project ECHO model. Participants represented diverse institutions, disciplines, and career stages, engaged voluntarily, and participated amid competing professional demands. Despite these less controlled conditions, the programs produced gains in self-reported knowledge, demonstrated intention to apply learning, and improved participants' perceived performance, skills and team-based communication and collaboration. At the same time, participants' reports of barriers—particularly limited time, resources, and the need for additional training—underscore the realities of real-world implementation and may help explain why effect sizes for skills and performance were smaller than those for knowledge.

Interpreting the evaluation findings through the TPB provides a useful lens for understanding how knowledge gains, intentions, and environmental constraints influence mentoring behavior among faculty. According to this theory, behavioral intentions arise from attitudes toward the behavior, subjective norms, and perceived behavioral control (PBC).^22^ The substantial increases in self-reported knowledge across both mentoring programs suggest more positive attitudes toward applying mentoring practices, as participants perceived the content as highly relevant and beneficial to their professional work. Improvements in these attitudinal components have been shown to strengthen behavioral intentions in diverse academic and health-related contexts.^25, 26^

Participants reported strong intentions to apply mentoring skills in both individual and team-based settings. This intention aligns with TPB research, which shows that intentions are the most immediate precursor to behavior.^22^ However, extensive evidence indicates that even strong intentions do not always translate directly into action, creating what has been termed the “intention—behavior gap”.^27–29^ This phenomenon is especially common in complex professional environments where multiple external demands compete for time and attention.

The barriers described by participants—particularly lack of time, insufficient resources, and the need for additional training—map directly onto the construct of perceived behavioral control. PBC reflects both confidence in performing the behavior and the extent to which external conditions facilitate or impede action.^30^ These barriers indicate that even when faculty members are motivated to engage in effective mentoring, structural constraints may limit the degree to which they can carry out their intentions. This interpretation is consistent with research showing that PBC contributes independently to behavior prediction and often moderates the strength of the intention—behavior relationship.^25^

Beyond individual-level determinants, organizational context may play an important role in shaping both perceived control and behavioral follow-through. Recent research emphasizes that mentoring behaviors may be more likely to develop in environments where organizations appear to cultivate a supportive mentoring climate—through elements such as explicit expectations, recognition systems, accessible mentoring tools, and leadership engagement.^8, 9, 31, 32^ Such climates may help enhance faculty members' sense of controllability by potentially providing time, resources, and social reinforcement for mentoring. When organizational culture seems to legitimize mentoring as essential academic work rather than an optional or “extra” responsibility, individuals may experience fewer barriers and may find more opportunities to practice mentoring behaviors.^31^

The multi-institutional reach of these programs, spanning professions and geographic regions, may also help strengthen subjective norms by exposing participants to peers who value and practice effective mentoring. Although subjective norms were not directly measured in this evaluation, gains in teamwork-related items suggest that the social environment within professional communities may be shifting toward greater alignment with mentoring best practices.

Finally, early behavioral impacts—such as improvements in communication, collaboration, and some performance indicators—suggest that participants are beginning to bridge the gap between intention and action. However, the modest size of these performance changes relative to knowledge and intention gains indicates that additional institutional supports may be required. Evidence from TPB-based intervention research suggests that targeted strategies—such as structured planning, protected time, and enhanced resource availability—can improve the translation of intention into sustained behavior.^27, 30^

A key strength of this evaluation is its broad scope: the programs delivered a high volume of sessions over multiple cohorts, engaged faculty from diverse professional backgrounds and institutions, and produced consistent improvements in knowledge, intentions, and team-related competencies. From a TPB perspective, another advantage is the inclusion of measures that correspond to central constructs—specifically behavioral intentions and perceived behavioral control—which allows the findings to be interpreted through an established theoretical lens. Our structured programs are innovative, cost-effective, practical, realistic, scalable, and sustainable across a broad range of institutions. The programs use the Project ECHO platform, which has international reach and is easily transferable and scalable. Over 5350 peer-reviewed publications testify to ECHO's advantages in trust, credibility, consistency, and replicability. ECHO utilizes an effective replication program to scale the model globally, maintain fidelity, and be responsive to regional needs. The US Senate's ECHO 2016 Act (S.2873) advocates using the ECHO model due to its scalability advantage for underserved areas. The ECHO infrastructure has been replicated in over 9000 cities in 180 countries (Figure 3). Our proposed ECHO-based curriculum is thus transferable to institutions worldwide.

The evaluation also has several limitations. Response rates to the surveys were modest (21–38%), raising the possibility that the results may not fully represent all participants' experiences. Because outcomes were self-reported, the findings may reflect participants' perceptions more than their actual behaviors. In addition, the analysis relied on unpaired pre- and post-data due to the absence of individual-level linkage, preventing within-person causal inference.

## Conclusions

In summary, viewing the program outcomes through the TPB framework highlights how knowledge gains, positive attitudes, supportive norms, and strong intentions set the stage for behavior change, while structural barriers shape the extent to which those intentions can be realized. The presence of environmental constraints—particularly those related to time, resources, and institutional expectations—underscores the importance of strengthening organizational systems that enhance perceived behavioral control. Creating a mentoring climate that values, supports, and rewards mentoring work may be essential for helping faculty move from intention to consistent practice. These findings suggest that high-quality mentoring programs, when paired with organizational environments that reduce barriers and reinforce mentoring as core academic work, have the potential to foster more sustainable mentoring behaviors across academic health settings.

## Supplementary Material

1

## Figures and Tables

**Figure 1:**
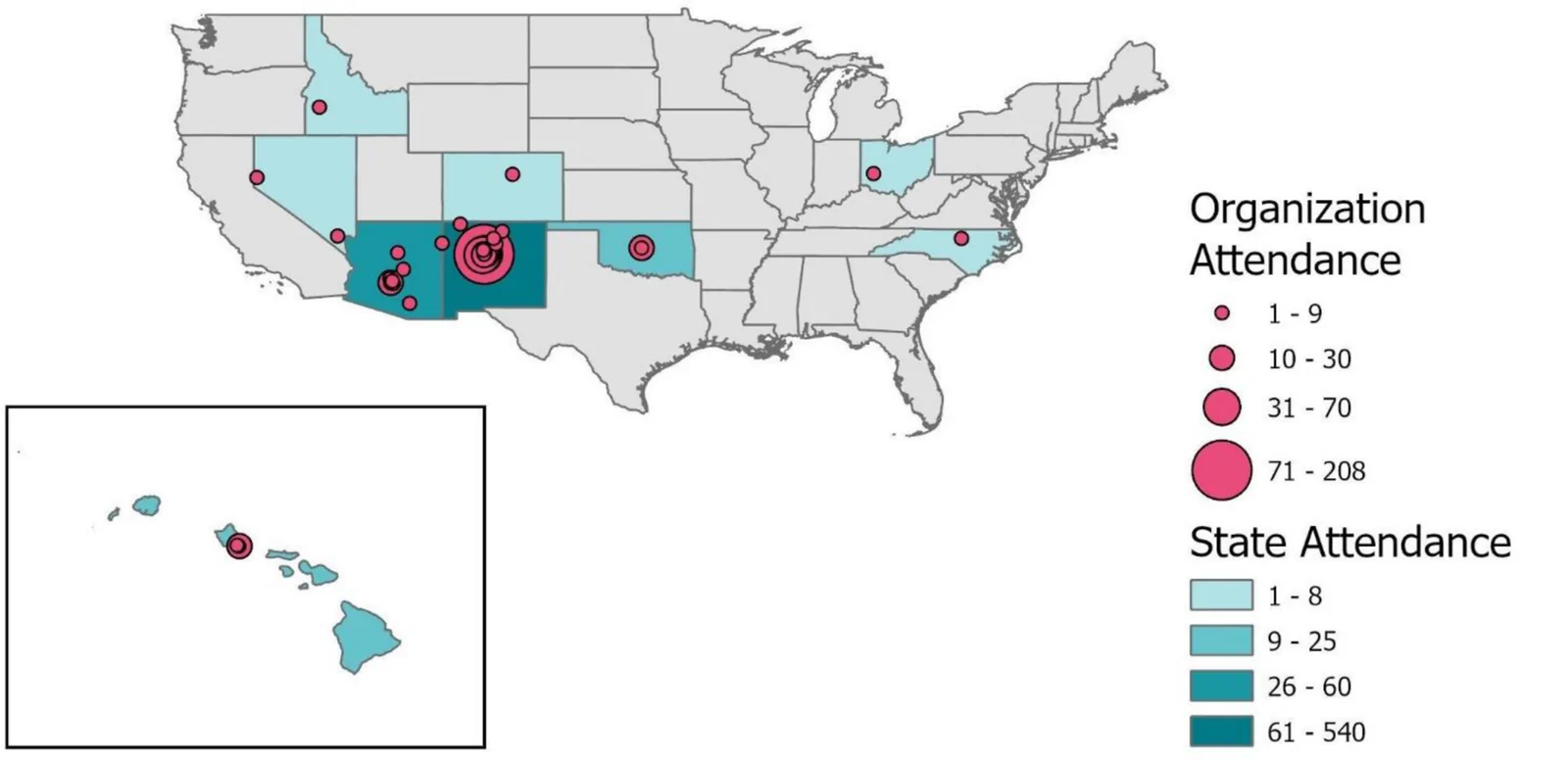
Bivariate map of the continental United States and Hawaii, showing the Mentor Development ECHO-based program attendees by organization and state, during the study timeframe. Organizations at each location are represented by graduated red circles, with the circle's diameter indicating total attendance within the organization. Concentric circles show multiple organizations at the same location. State attendance is shown on a choropleth map, with shades of color indicating the level of attendance.

**Figure 2:**
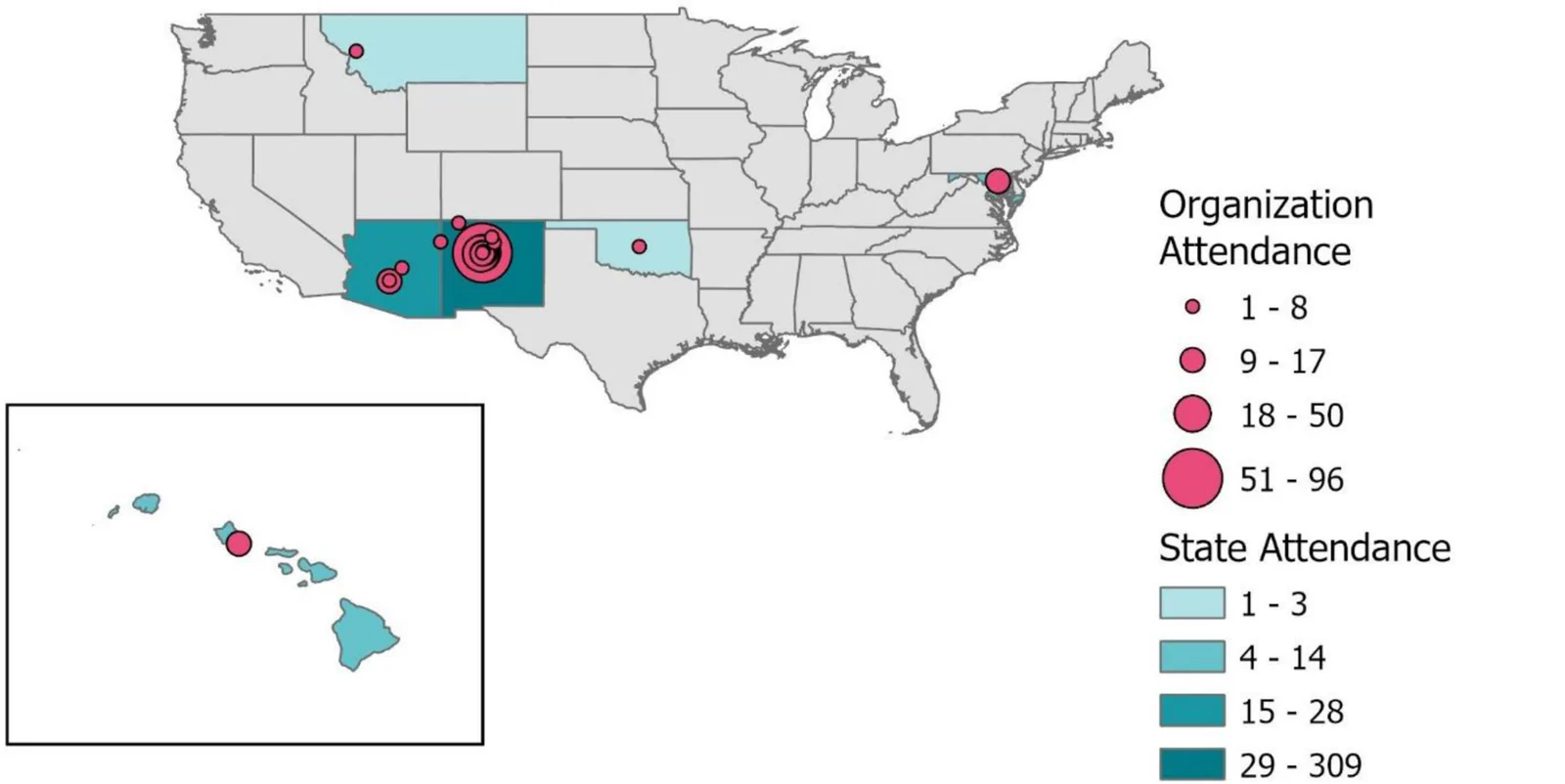
Bivariate map of the continental United States and Hawaii, showing Mentoring Networks ECHO-based program attendees by organization and state, during the study timeframe. Organizations at each location are represented by graduated red circles, with the circle's diameter indicating total attendance within the organization. Concentric circles show multiple organizations at the same location. State attendance is shown on a choropleth map, with shades of color indicating the level of attendance.

**Figure 3:**
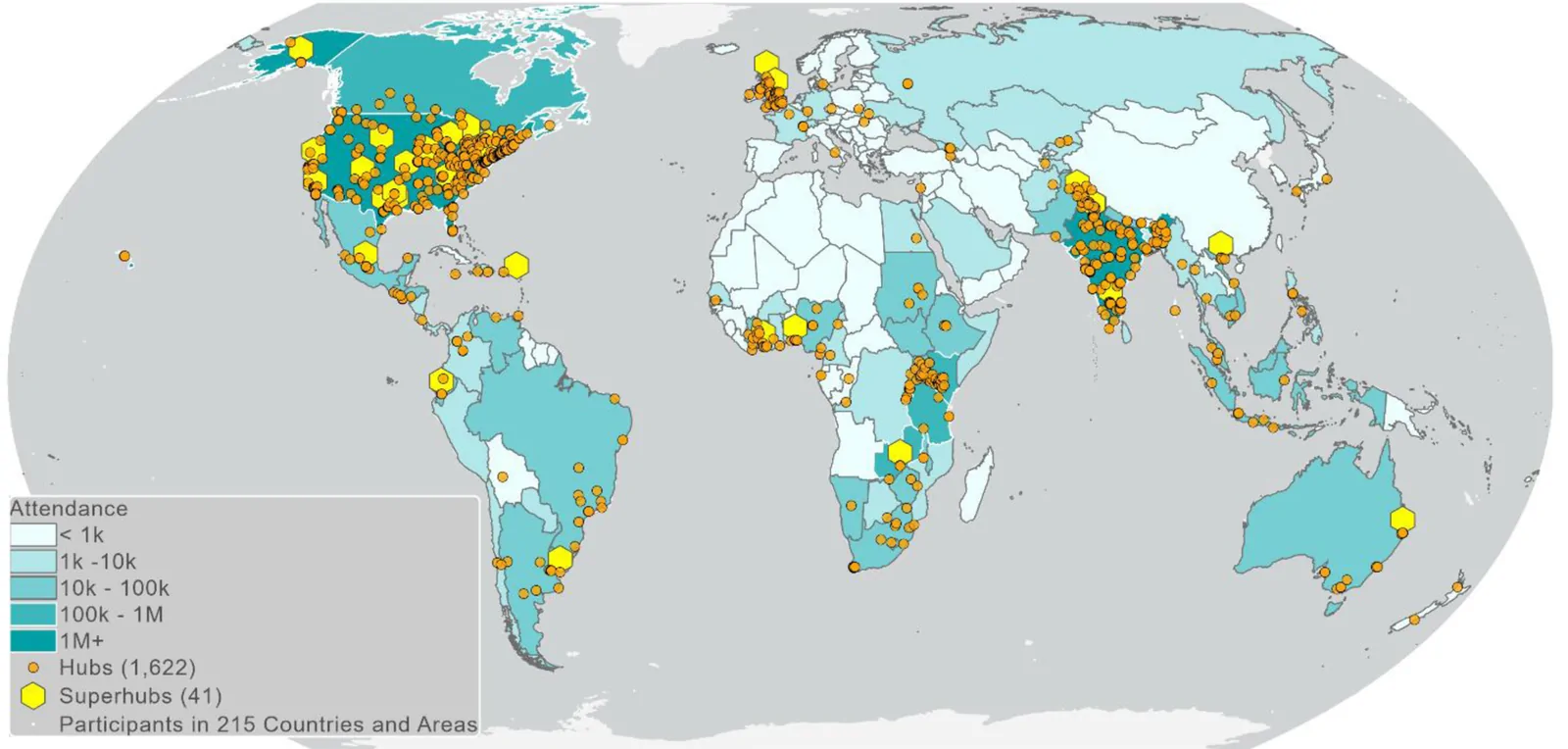
Map of global ECHO hubs demonstrates reach of the telementoring model & potential for scalability of our intervention.

**Table 1:** Curricular Components of the Faculty Mentor Development Program

Module number	Module	Objectives
1	Defining Mentoring from the Beginning	Define and describe mentoring, including the origin of the concept Describe the various roles played by mentors of junior faculty in academic research Map mutual relationships, expectations, and responsibilities in the mentoring relationship Develop appropriate mentoring plans
2	Rewards and Challenges of Mentoring	Describe the evidence that suggests the need for effective mentoring in biomedical research Discuss the rewards of becoming a mentor Consider effective mentoring challenges Discuss effective ways to end unsuccessful mentoring relationships Discuss case scenarios that illustrate strategies for meeting mentoring challenges
3	Communicating Effectively with Mentees	Evaluate communication styles and recognize the pros and cons of different styles Describe active listening and its importance in effective communication Identify ways to effectively convey your ideas Identify ways to promote mentee communication and self-efficacy Define emotional intelligence and its importance in effective communication
4	Achieving Work-Life Balance	Define work-life or personal-professional balance and its importance Identify characteristics of “burnout” Identify strategies to prioritize tasks and avoid “burnout”
5	Understanding Diversity Among Mentees	Explain why diversity is important for excellence Describe existing faculty diversity and initiatives to enhance diversity Define unconscious bias Identify strategies to overcome unconscious bias and help the mentee succeed across differences
6	Benefits of Formal Mentoring and Informal Mentoring Relationships	Define informal mentoring Discuss the process of informal mentoring, including the roles and responsibilities of mentoring partners Compare informal mentoring with formal mentoring
7	Leadership Skills and Opportunities	Define leadership and situational leadership Define and distinguish between leading and managing, and explore the practical applications of both Describe motivating factors, and understand those factors that lead to satisfaction or dissatisfaction Identify your strengths and weaknesses in leading, both one-on-one and in team situations
8	Helping Mentees Get and Manage Funding	Identify internal and external funding sources for mentees Identify components for successful management of grant awards Identify appropriate strategies for interacting with IRB and rebounding after grant rejection

**Table 2:** Curricular Components of the Mentoring Networks Program

Module number	Module name	Module objective
1	Social Networks: Importance of Connections	Define Social Networks Identify its structural properties Explain the importance of social networks in creating and fostering connections
2	Developmental Networks	Describe benefits and properties of a developmental network Apply network concepts to support career development of junior faculty
3	Developmental Networks – Structure and Content	Differentiate roles of participants in a developmental network Distinguish key resources and support provided by participants in a developmental network Evaluate strategies to enlist relevant developers
4	Developmental Network Analysis	Review your developmental network map Determine the structure, content, and diversity of your current developmental network
5	Building Your Development Network	Identify gaps in your developmental network based on your career goals Formulate an action plan for improving your developmental network
6	High-Quality Connections	Identify the characteristics of high-quality connections Develop strategies to cultivate these connections
7	Team Dynamics in Facilitated Group Mentoring	Describe strategies for facilitating healthy group interactions Discuss approaches for preventing destructive conflict in groups Apply techniques for dealing with conflict in a case study
8	Networking in Professional Settings	Describe strategies for professional networking Discuss how to approach networking if you are an introvert

**Table 3:** Session Attendance and Evaluation Response Rates for Mentor Development and Mentoring Networks Programs

		Per Timeframe			Per Session	
Program	Month and Year	Number of Sessions for the Timeframe	Total Attendances (not unique individuals)	Total Evaluation Responses for the Timeframe (not unique individuals)	Average Attendance Per Session for the Timeframe (unique individuals)	Evaluation Response Rate Per Session for the Timeframe (unique individuals)
Mentor Development	Aug-23	5	125	44	25.0	35.2%
Mentor Development	Sep-23	3	54	24	18.0	44.4%
Mentor Development	Mar-24	4	85	34	21.3	40.0%
Mentor Development	Apr-24	4	68	30	17.0	44.1%
Mentor Development	Aug-24	4	120	59	30.0	49.2%
Mentor Development	Sep-24	4	99	44	24.8	44.4%
Mentor Development	Mar-25	4	60	14	15.0	23.3%
Mentor Development	Apr-25	1	15	5	15.0	33.3%
Mentor Development	Apr-25	3	38	10	12.7	26.3%
Mentor Development	Aug-25	4	90	28	22.5	31.1%
Mentor Development	Sep-25	4	67	18	16.8	26.9%
**Mentor Development-Totals**	**Aug 23 – Sep 25**	**40**	**821**	**310**	**20.5**	**37.8**
Mentoring Networks	Oct-23	4	75	10	18.8	13.3%
Mentoring Networks	Nov-23	4	71	18	17.8	25.4%
Mentoring Networks	May-24	5	63	11	12.6	17.5%
Mentoring Networks	Jun-24	3	35	4	11.7	11.4%
Mentoring Networks	Oct-24	5	93	25	18.6	26.9%
Mentoring Networks	Nov-24	3	55	11	18.3	20.0%
Mentoring Networks	May-25	4	27	2	6.8	7.4%
Mentoring Networks	Oct-25	5	87	25	17.4	28.7%
Mentoring Networks	Nov-25	3	50	10	16.7	20.0%
**Mentoring Networks-Totals**	**Oct 23 – Nov 25**	**36**	**556**	**116**	**15.4**	**21.0%**

Note 1: Mentoring Network courses in June 2025 were abandoned after four sessions due to inadequate attendance

**Table 4:** Change in Knowledge Scores for Mentor Development and Mentoring Networks ECHO-based Programs

Program	Status	Not at all knowledgea ble Or 1	Slightly knowledgea ble Or 2	Moderately knowledgea ble Or 3	Very knowledgea ble Or 4	Extremely knowledgea ble Or 5	Mean	SD	P- value
		n (%)	n (%)	n (%)	n (%)	n (%)			
Mentor Development (N=314)	Pre-test	25 (8%)	130 (41%)	122 (39%)	31 (10%)	6 (2%)	2.6	0.85	<0.001
Mentor Development (N=314)	Post-test	0 (0%)	15 (5%)	120 (38%)	150 (48%)	29 (9%)	3.6	0.72	
Mentoring Networks (N=124)	Pre-test	15 (12%)	65 (52%)	41 (33%)	1 (1%)	2 (2%)	2.3	0.75	<0.001
Mentoring Networks (N=124)	Post-test	1 (1%)	8 (6%)	49 (40%)	64 (52%)	2 (2%)	3.5	0.68	

Note 1: Pre-test refers to retrospective pre-test data collection

Note 2: Unpaired t-test comparison was performed within each program, due to the lack of individual-level data.

Note 3: Using conventional benchmarks for Cohen’s d (≈0.2 small, 0.5 medium, 0.8 large), both programs show very large pre→post gains on the 1–5 knowledge scale; Mentoring Networks’ standardized gains are somewhat larger than Mentor Development program’s (Cohen’s d of 1.7 vs. 1.3, respectively).

**Table 5:** Analysis of Post-Test Questions on Competence and Performance for Mentor Development and Mentoring Networks ECHO-Based Programs

Program	Question		1	2	3	4	5	
		N	n (%)	n (%)	n (%)	n (%)	n (%)	Mean (SD)
			Definitely not	Probably not	Possibly	Probably yes	Definitely yes	
**Mentor Development**	Will you use what you learned in this session in your work?	314	1 (0%)	4 (1%)	32 (10%)	39 (12%)	236 (75%)	4.6 (0.75)
**Mentoring Networks**	Will you use what you learned in this session in your work?	124	0 (0%)	3 (2%)	10 (8%)	26 (21%)	85 (69%)	4.6 (0.75)
			Not at all relevant	Slightly relevant	Moderately relevant	Very relevant	Extremely relevant	
**Mentor Development**	How relevant is this session to your current work?	210	3 (1%)	13 (6%)	41 (20%)	85 (40%)	68 (32%)	4.0 (0.95)
**Mentoring Networks**	How relevant is this session to your current work?	90	2 (2%)	8 (9%)	14 (16%)	43 (48%)	23 (26%)	3.9 (0.98)
			Strongly disagree	Disagree	Neutral	Agree	Strongly agree	
**Mentor Development**	I intend to apply the knowledge/skills in a team environment.	314	5 (2%)	3 (1%)	26 (8%)	106 (34%)	174 (55%)	4.4 (0.81)
**Mentoring Networks**	I intend to apply the knowledge/skills in a team environment.	124	0 (0%)	1 (1%)	7 (6%)	47 (38%)	69 (56%)	4.5 (0.64)
**Mentor Development**	I am better able to communicate/collaborate in multidisciplinary teams.	314	5 (2%)	6 (2%)	46 (15%)	117 (37%)	140 (45%)	4.2 (0.88)
**Mentoring Networks**	I am better able to communicate/collaborate in multidisciplinary teams.	124	0 (0%)	1 (1%)	15 (12%)	44 (35%)	64 (52%)	4.4 (0.73)

**Table 6:** Self-Reported Impact by Program for Mentor Development and Mentoring Networks ECHO-Based Programs

This session has increased, improved, or positively impacted my:	Mentor Development (Counts, %)	Mentoring Networks (Counts, %)

Knowledge	263 (84%)	117 (94%)
Skills	185 (59%)	76 (61%)
Performance	102 (32%)	45 (36%)
No Change	18 (6%)	3 (2%)

**Table 7:** Self-Reported Barriers for Mentor Development and Mentoring Networks ECHO-Based Programs

Barrier factor	Mentor Development (n, % of respondents)	Mentoring Networks (n, % of respondents)

There are no barriers to using what I learned	201 (64%)	53 (43%)
Need more training	62 (20%)	25 (20%)
Lack of time	44 (14%)	42 (34%)
No opportunities to apply in my work	18 (6%)	11 (9%)
Lack of resources	18 (6%)	16 (13%)
Other	9 (3%)	6 (5%)
