# Supplementary material for: A scalable dissemination model for training faculty in mentor development and networking
Source: Med Res Arch. Author manuscript; Available in PMC 2026 Oct 6. (PMC13637551; doi:10.18103/mra.v14i5.7423)
Supplement: Supplementary file 1 [file NIHMS2196032-supplement-1.pdf]

APPENDIX

Table S1: Professions Breakdown for Attendees of the Mentor Development and Mentoring Networks ECHO Programs

	Mentor Development		Mentoring Networks	
**Profession**	**Unique individuals (n)**	%	**Unique Individuals (n)**	%
Physician-MD/DO	185	23%	118	21%
Nurse/nurse practitioner	81	10%	43	8%
Pharmacist	34	4%	18	3%
Social Worker	8	1%	1	0%
Registered Dietician	8	1%	7	1%
PhD & Other/Unknown	501	61%	369	66%
TOTAL	821		556	

Note: Other category included non-clinicians, e.g., PhD faculty

Table S2: Credits Awarded for Mentor Development and Mentoring Networks ECHO Programs

	**Mentor Development**		**Mentoring Networks**	
**Education Credit Type Awarded**	**Attendees requesting credit (not unique individuals)**	**Hours of credits**	**Attendees requesting credit (not unique individuals)**	**Hours of credits**
Continuing Medical Education (CME)	133	131.5	73	72.5
Continuing Education Units (CEU)	38	38	10	9.5
Continuing Education (CE)	32	32	6	5.5
Continuing Nursing Education (CNE)	35	34.5	4	4
Grand Totals	238	236	93	91.5
